# Integrative metagenomic and metabolomic profiling identifies gut microbial and metabolite signatures associated with lymph node metastasis in pancreatic cancer

**DOI:** 10.3389/fmicb.2025.1706084

**Published:** 2025-12-12

**Authors:** Pengyu Li, Mingfei Wang, Hanyu Zhang, Xingyu Gao, Lixin Chen, Haomin Chen, Qiang Xu, Weijie Chen, Wenjing Liu, Menghua Dai

**Affiliations:** Department of General Surgery, Peking Union Medical College Hospital (PUMCH), Peking Union Medical College and Chinese Academy of Medical Sciences, Beijing, China

**Keywords:** pancreatic cancer, gut microbiome, gut metabolites, lymph node metastasis, predictive model

## Abstract

**Background:**

Lymph node metastasis (LNM) is a prognostic factor in pancreatic cancer. The association between the gut microbiota and LNM remains unexplored. This study aimed to characterize the gut microbiota and metabolomic profiles associated with LNM and to investigate their potential as predictive biomarkers.

**Methods:**

Fecal samples from pancreatic cancer patients undergoing surgery were analyzed using metagenomic sequencing and untargeted metabolomics. The patients were categorized into LNM and non-LNM (NLNM) groups. Differential microbiome taxa were analyzed using the DESeq2 package. Random forest predictive models were developed based on metagenomic and metabolomic data, with performance assessed using leave-one-out cross-validation (LOOCV).

**Results:**

A total of 26 patients with LNM and 29 patients without LNM were included. Principal coordinates analysis (PCoA) revealed significant differences in microbiota composition between the two groups (Anosim, *p* = 0.047). The absolute counts of *Ruminococcus gnavus* and *Blautia wexlera* were significantly decreased in LNM. Tryptophan-derived metabolites, indole-3-lactic acid (3-ILA) and indole-3-acrylic acid (3-IA), were downregulated in LNM. Functional pathway analysis showed downregulation of tryptophan metabolism in LNM, while cancer-related pathways were upregulated. Correlation analysis revealed a significant positive association between *Ruminococcus gnavus* and 3-ILA/3-IA levels. Moreover, *Ruminococcus gnavus* was positively correlated with CD8^+^ T cells. Predictive models based on the gut microbiota and metabolites distinguished LNM from NLNM, with AUC values of 0.854 and 0.940, respectively.

**Conclusion:**

The gut microbiota and metabolites exhibit significant alterations during lymph node metastasis in pancreatic cancer, especially *Ruminococcus gnavus*, *Blautia wexlera*, and tryptophan metabolites (3-ILA and 3-IA). Gut microbial and metabolite signatures may serve as potential non-invasive biomarkers for predicting LNM in pancreatic cancer. Further functional validation is required to determine whether and how the gut microbiota and metabolites may mediate lymph node metastasis.

## Introduction

Pancreatic cancer is one of the most aggressive malignancies of the digestive system, characterized by an insidious onset, early lymph node metastasis (LNM), poor prognosis, and resistance to chemotherapy and radiotherapy (Cui et al., 2024). The five-year survival rate remains below 13% (Siegel et al., 2024). Studies have shown that most patients with pancreatic cancer have already developed LNM by the time of diagnosis (Burke et al., 2015), and LNM is recognized as an independent prognostic factor affecting patient survival (Chun et al., 2018; Strobel et al., 2022; Lahat et al., 2016). Accurate preoperative assessment of lymph node metastasis is critical for guiding neoadjuvant therapy, informing subsequent treatment strategies, and improving postoperative outcomes. However, current diagnostic tools for predicting LNM are limited and mostly invasive. CA19-9, a specific biomarker for pancreatic cancer, shows only modest predictive performance for LNM (AUC = 0.653) (Tang et al., 2025). Combining CA19-9 with MRI radiomics features improves predictive efficiency to 0.740 (Tang et al., 2025), and CT-based radiomics achieves an AUC of 0.71–0.815 (Bian et al., 2022; Chen et al., 2023; Fu et al., 2023). Nevertheless, these approaches are limited by invasiveness, cost, and restricted generalizability. Therefore, it is crucial to explore novel non-invasive strategies for preoperative prediction of lymph node metastasis, to elucidate the underlying biological mechanisms, and to identify effective therapeutic targets for pancreatic cancer.

The gut microbiota is the largest microbial ecosystem in the human body, comprising bacteria, fungi, and viruses that play essential roles in maintaining host homeostasis, regulating energy metabolism, and modulating immune responses (Rooks and Garrett, 2016). Gut microbiota dysbiosis has been implicated in cancer development and progression by influencing metabolic and immune functions. Preclinical studies suggest that the gut microbiota may promote tumor progression by suppressing innate immunity (Thomas et al., 2018; Yu et al., 2022). In addition, the microbiota can activate pattern recognition receptors (PRRs), recruit adaptor proteins such as MyD88 and TRIF, and enhance MAPK and NF-κB signaling pathways, which may cooperate with the K-Ras pathway to drive pancreatic cancer progression (Ochi et al., 2012; Zambirinis et al., 2013). Furthermore, depletion of the gut microbiota has been shown to enhance the immunogenicity of pancreatic cancer by promoting Th1 polarization of CD4^+^ T cells, increasing CD8^+^ T cell infiltration, and reducing myeloid-derived suppressor cell (MDSC) accumulation (Pushalkar et al., 2018). Other studies have revealed that the gut microbiota can facilitate tumor progression by modulating the pancreatic tumor microenvironment and reducing the infiltration and cytotoxic activity of natural killer (NK) cells (Yu et al., 2022). In addition, gut microbiota-derived 3-indoleacetic acid (3-IAA) can elevate reactive oxygen species (ROS) levels in pancreatic cancer, thereby improving the efficacy of chemotherapy (Tintelnot et al., 2023). Collectively, these findings highlight the close association between the gut microbiota and the initiation and progression of pancreatic cancer. Given its accessibility and stability, the gut microbiome represents a promising source of non-invasive biomarkers. Our previous research demonstrated that gut microbial signatures can serve as accurate diagnostic markers for pancreatic cancer (Li et al., 2025). Moreover, the gut microbiota has been reported to predict distant metastasis in patients with pancreatic cancer (Villani et al., 2024). Therefore, constructing predictive models based on gut microbial features may provide new avenues for the early and accurate prediction of lymph node metastasis in pancreatic cancer.

This study aimed to characterize the differences in gut microbial composition between pancreatic cancer patients with and without LNM and to identify key microbial taxa and metabolites potentially associated with LNM. Furthermore, we sought to develop non-invasive predictive models based on microbiome and metabolite profiles for LNM to facilitate preoperative identification of high-risk patients, ultimately optimizing clinical decision-making. In addition, previous studies have shown significant differences in the oral microbiome between pancreatic cancer patients and healthy controls (Kartal et al., 2022). Therefore, we also aimed to investigate whether the oral microbiome undergoes changes during the process of LNM in pancreatic cancer.

## Materials and methods

### Participants and study design

Participants were prospectively recruited between May 2021 and September 2024 at Peking Union Medical College Hospital (PUMCH). Individuals aged 18 to 75 years were considered eligible for inclusion, but they were excluded if they met any of the following criteria: (1) A prior diagnosis of other malignancies, infectious diseases, psychiatric or neurodegenerative disorders, or conditions affecting the oral or gastrointestinal systems and (2) recent medical treatments or procedures within specific time frames, including: (a) Use of antibiotics, hormone therapy, or immunosuppressive agents within the past 3 months; (b) gastrointestinal reconstructive surgery within the past 3 months; (c) regular use of laxatives, antidiarrheal medications, or high-dose probiotics within the past month; and (d) participation in gastrointestinal examinations within the past 3 days. All participants underwent radical surgery, with standard lymph node dissection performed according to the ISGPS guidelines (Tol et al., 2014). Pancreatic ductal adenocarcinoma (PDAC) was histologically confirmed using resected specimens. Based on lymph node metastasis status, the participants were categorized into two groups: the lymph node metastasis (LNM) group and the non-lymph node metastasis (NLNM) group. This study was approved by the Peking Union Medical College Hospital Institutional Review Board (K7866) and adhered to the principles of the Declaration of Helsinki. Informed consent was obtained from all patients prior to sample collection.

### Sample collection

Fecal and oral sample collection methods have been described previously (Li et al., 2025; Li et al., 2024). All samples were immediately placed in cryopreservation containers upon collection and stored at −80 °C within 1 h for further analysis.

### DNA extraction, 16s rRNA gene amplicon sequencing, metagenomic sequencing, and data processing

Since the oral samples were collected using swabs, the bacterial quantity was only sufficient for 16S rRNA sequencing. In contrast, the fecal samples were subjected to metagenomic sequencing. The procedures for DNA extraction, 16S rRNA sequencing, and library construction of the oral samples have been described previously (Li et al., 2025; Li et al., 2024). For 16S rRNA gene sequencing, paired-end reads were quality-filtered, merged, and denoised using DADA2 to obtain amplicon sequence variants (ASVs). Sequence alignment was performed using BLAST, and representative sequences were annotated using the SILVA database (Quast et al., 2013). Alpha and beta diversity were calculated using QIIME2 and visualized using the R package.

For metagenomic analysis, metagenome libraries were sequenced on an Illumina NovaSeq 6000 platform (PE150) at LC-Bio Technology Co., Ltd. (Hangzhou, China). After quality control, host-derived reads were removed by aligning to the human reference genome (GRCh38). The quality-filtered reads were *de novo* assembled to construct metagenomes, and coding regions (CDS) of metagenomic contigs were predicted and clustered to obtain unigenes (MetaGeneMark, CD-HIT). Taxonomic and functional annotation of the unigenes was performed against the NCBI NR database using DIAMOND (v0.9.14) (Buchfink et al., 2015). Differential abundance of the unigenes between the two groups was analyzed using DESeq2 (Riquelme et al., 2019). DESeq2 is a method for differential abundance testing of sequence data based on a negative binomial distribution model and is particularly sensitive to small sample sizes and subtle differences in library sizes (Love et al., 2014). Differential abundance was defined as having an adjusted *p*-value (Benjamini–Hochberg corrected) of < 0.05. In addition, linear discriminant analysis (LDA) effect size (LEfSe) was performed to examine inter-group significance and biological differences at various taxonomic levels, with a threshold of LDA > 3.0 and a *p*-value of < 0.05.

### Metabolomics analysis and data analysis

Metabolites were extracted from the fecal samples with an 80% methanol buffer and incubated at 24 °C for 10 min. After centrifugation at 4,000×*g* for 20 min at 4 °C, the supernatants were subjected to liquid chromatography–mass spectrometry (LC–MS) analysis. Chromatographic separation was performed on an ultra-performance liquid chromatography (UPLC) system (SCIEX, UK) equipped with an ACQUITY UPLC HSS T3 column (100 mm × 2.1 mm, 1.8 μm, Waters, UK). Metabolites were detected using a Q Exactive high-resolution mass spectrometer (Thermo Fisher Scientific, Bremen, Germany) operated in both positive ion mode (PIM) and negative ion mode (NIM).

The raw LC–MS data were processed using XCMS and the metaX software package (Smith et al., 2006; Wen et al., 2017). Metabolites were annotated based on accurate mass, MS/MS fragment spectra, and isotope ratio differences using the Human Metabolome Database (HMDB)^1^ and KEGG.^2^ According to the Metabolomics Standards Initiative (MSI), all annotated metabolites were considered putatively identified (Level 2). Metabolite features were log₂-transformed prior to statistical analysis. Partial least squares–discriminant analysis (PLS-DA) was conducted to visualize metabolic differences between the groups. Differential metabolites were identified based on a VIP ≥ 1, |fold change (FC)| ≥ 1.2, and *p*-value < 0.05 (Student’s *t*-test). The annotated metabolites were further mapped to the KEGG and HMDB databases for pathway identification. Functional enrichment analysis was conducted using Gene Set Enrichment Analysis (GSEA, v4.1.0) with MSigDB reference gene sets, and pathways with a |normalized enrichment score (NES)| > 1, nominal *p*-value < 0.05, and FDR < 0.25 were considered significantly enriched.

### Lymphocyte immunophenotyping

Peripheral whole blood was collected in EDTA tubes before surgery and analyzed using antibodies targeting CD3/CD8/CD4, CD3/CD16CD56/CD19, and isotype controls (Immunotech, France). Lymphocyte subset counts were determined using a dual-platform method based on white blood cell counts and lymphocyte differentials from routine blood tests.

### Predictive classifier establishment

A random forest algorithm was used to construct predictive classifiers for lymph node metastasis, based on the top 20 discriminatory markers identified separately from fecal microbiome and metabolomic data. Leave-one-out cross-validation (LOOCV) was used to evaluate performance and robustness. This process was repeated for each sample in the dataset, ensuring that each observation served as a test instance once. The area under the receiver operating characteristic curve (AUC) was calculated to assess classifier performance.

### Statistical analysis

Quantitative data were expressed as mean ± standard deviation (SD) or median [interquartile range (IQR)]. The independent samples *t*-test or Mann–Whitney U test was used for comparisons, as appropriate. Categorical data were presented as frequencies and percentages, and comparisons were performed using Pearson’s chi-squared test, continuity correction, or Fisher’s exact test. All statistical analyses were conducted using the R software (v.4.4.0). In the network analysis, microbial co-occurrence networks were inferred using Spearman’s rank correlations and visualized with the Gephi software (v0.10.1), with a correlation magnitude threshold of 0.6 and a *p*-value of 0.05. Random forest classification was performed using the randomForest R package (v.4.7.1.1). Other R packages applied in the study included the following: ggplot2 (v.3.5.1), metagenomeSeq (v.1.38.0), car (v.3.1.2), vegan (v.2.6.10), corrplot (v.0.95), OmicStudioClassic (v.1.74.0), OmicStudioKits (4.3.0), metaX (v. 2.0.0), pROC (v.1.18.5), caret (v.6.0.94), VennDiagram (v.1.7.3), DESeq2 (v.1.46.0), readr (v.2.1.55), dplyr (v.1.1.4), and stats (v.4.0.3). Statistical significance was defined as a two-sided *p*-value of < 0.05.

## Results

### Characteristics and gut microbiome composition of the patients in the LNM and NLNM groups

After applying strict inclusion and exclusion criteria, fecal samples were collected from 26 patients with lymph node metastasis and 29 patients without lymph node metastasis. The two groups were comparable in terms of age, sex, tumor location, history of diabetes, and T stage (Table 1).

**Table 1:** Characteristics of the patients in the LNM and NLNM groups.

Variables	LNM (N = 26)	NLNM (N = 29)	P-value
Age (median (IQR), year)	58.5 (54.0, 62.0)	63.0 (58.0, 69.0)	0.070
Sex (male), *n* (%)	16 (61.5%)	17 (58.6%)	0.825
BMI (median (IQR), kg/m^2^)	22.95 (22.04, 24.21)	23.49 (21.34, 25.71)	0.462
Hypertension, *n* (%)	8 (30.8%)	10 (34.5%)	0.769
Diabetes, *n* (%)	9 (34.6%)	10 (34.5%)	0.992
Obstructive jaundice, *n* (%)	6 (23.1%)	2 (6.9%)	0.188†
Tumor location, *n* (%)			0.075
Head	17 (65.4%)	12 (41.4%)	
Body and tail	9 (34.6%)	17 (58.6%)	
History of smoking, *n* (%)	12 (46.2%)	7 (24.1%)	0.086
History of drinking, *n* (%)	9 (34.6%)	11 (37.9%)	0.799
CA19-9, U/mL	88.7 (36.3, 210.0)	38.7 (16.8, 150.0)	0.159
T stage			0.904
T1	6 (23.1%)	6 (20.7%)	
T2	15 (57.7%)	16 (55.2%)	
T3	5 (19.2%)	7 (24.1%)	
Perineural invasion, *n* (%)	22 (84.6%)	20 (69.0%)	0.173
Adipose tissue invasion, *n* (%)	23 (88.5%)	26 (89.7%)	1.000†
Resection margin, *n* (%)			0.335†
R0	21 (80.8%)	27 (93.1%)	
R1	5 (19.2%)	2 (6.9%)	

Categorical data are reported as numbers and frequencies. Continuous data are reported as median (IQR). LNM refers to lymph node metastasis. NLNM refers to non-lymph node metastasis. BMI, body mass index.

^†^Continuity correction. A two-sided *p*-value of < 0.05 was considered statistically significant.

**Figure 1:**
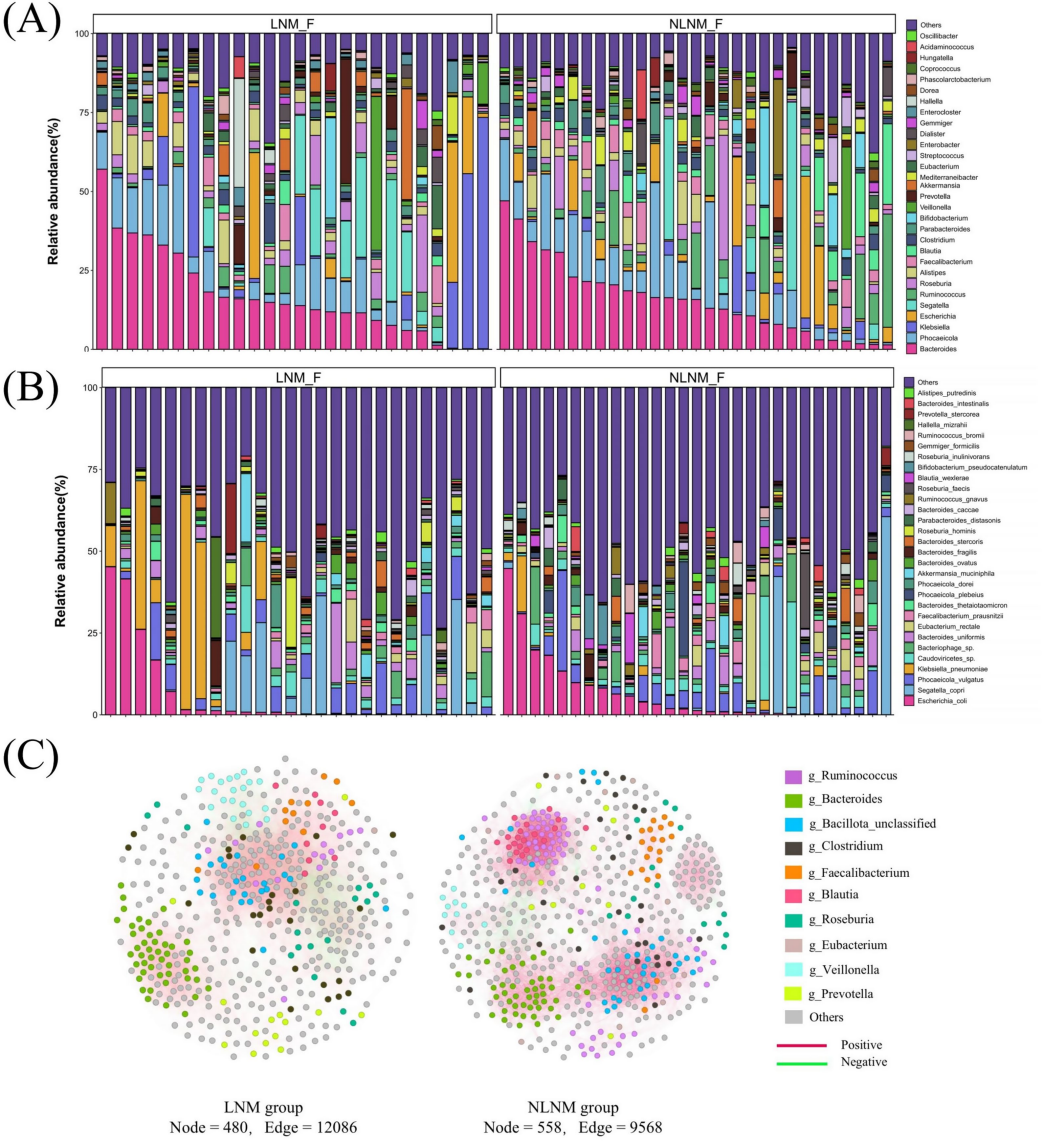
Top 30 most abundant fecal microbiota and microbial co-occurrence network in the LNM and NLNM groups. Genus level **(A)**; Species level **(B)**. LNM, lymph node metastasis; NLNM, non-lymph node metastasis. The microbial co-occurrence network was deduced using Spearman’s rank correlations based on the samples from the LNM and NLNM groups. Only statistically significant (*p* < 0.05) connections with magnitude > 0.6 (positive correlation, red edges) or < −0.6 (negative correlation, green edges) are shown. Each node represents a microbial species, and the color indicates taxonomic assignment to genera **(C)**.

A total of 8,072,780 unigenes were obtained from the 55 patient samples, identifying 3,340 genera and 18,660 species. Rarefaction curves based on the Shannon index reached a plateau in both groups, indicating sufficient sequencing depth (Supplementary Figure S1A). Distinct dominant microbial compositions were observed between the LNM and NLNM groups at both the genus and species levels. (Figures 1A,B). Both groups shared 156 phyla, 2,903 genera, and 15,181 species (Supplementary Figure S2). The microbial co-occurrence network revealed distinct clustering patterns between the two groups. In the NLNM group, members of *Ruminococcus* and *Bacteroides* formed relatively independent intra-genus clusters, indicating stronger within-genus associations. In contrast, the LNM group was characterized by clusters dominated by *Bacteroides* and *Veillonella* (Figure 1C).

### Comparison of gut microbiome composition in the LNM and NLNM groups

**Figure 2:**
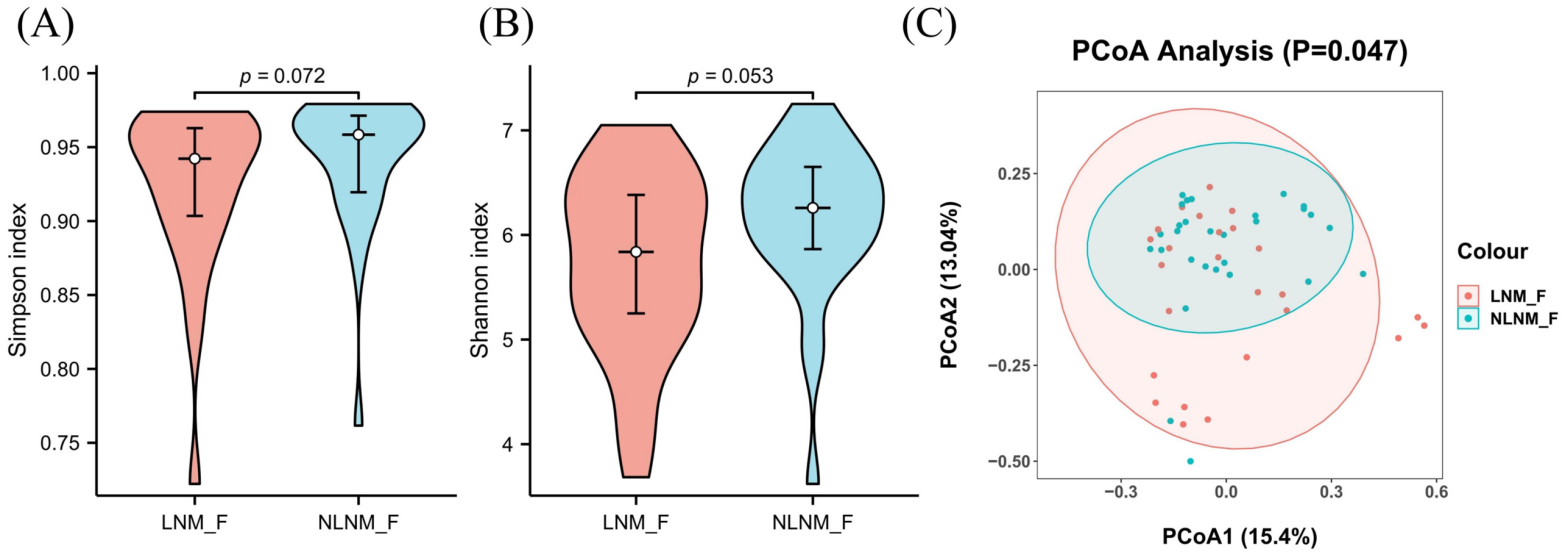
Comparison of microbial diversity between the LNM and NLNM groups. Regarding *α*-diversity, the Simpson **(A)** and Shannon indices **(B)** were slightly higher in the NLNM group compared to the LNM group, but the differences were not statistically significant (*p* = 0.072, *p* = 0.053). Bray–Curtis principal coordinates analysis (PCoA) revealed significant differences in the fecal microbiome between the LNM and NLNM groups (*p* = 0.047, R^2^ = 0.043) **(C)**. LNM, lymph node metastasis; NLNM, non-lymph node metastasis. A two-sided *p*-value of <0.05 was considered statistically significant.

The Simpson and Shannon indices were slightly higher in the NLNM group compared to the LNM group, but the differences were not statistically significant (*p* = 0.072, *p* = 0.053) (Figures 2A,B). Bray–Curtis principal coordinates analysis (PCoA) revealed a significant difference between the two groups (*p* = 0.047, R^2^ = 0.043) (Figure 2C).

**Figure 3:**
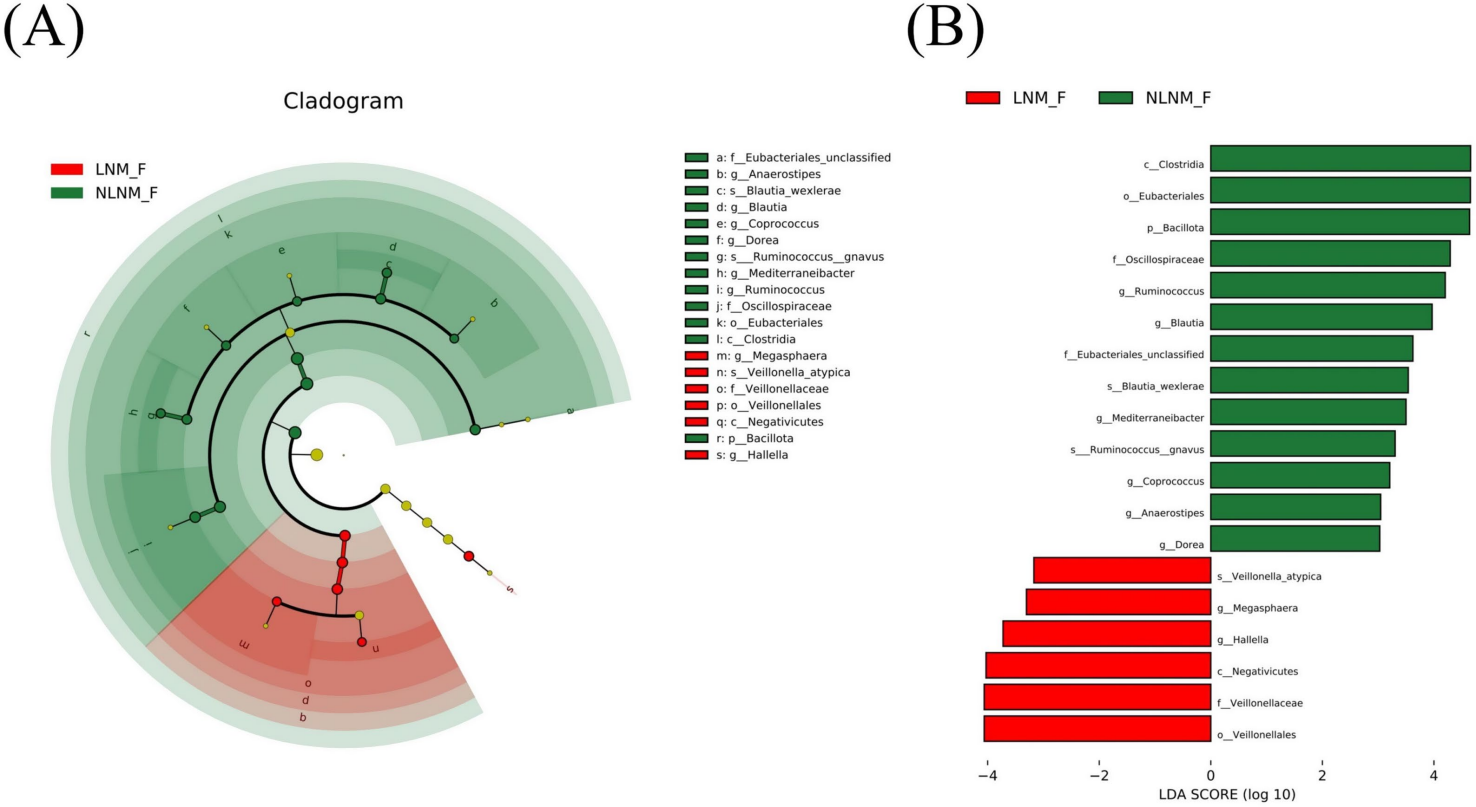
Differential genera and species between the LNM and NLNM groups. Differential taxa identified by LEfSe analysis: cladogram **(A)** and LDA score distribution bar plot **(B)**. LNM, lymph node metastasis; NLNM, non-lymph node metastasis; LDA, linear discriminant analysis.

Supplementary Tables 1,2 summarize all differential taxa between the LNM and NLNM groups at the genus and species levels, respectively, as identified by DESeq2 following Benjamini–Hochberg correction. At the species level, *Phocaeicola plebeius*, *Bifidobacterium pseudocatenulatum*, *Blautia wexlerae*, and *Ruminococcus gnavus* were significantly enriched in the NLNM group, whereas *Klebsiella pneumoniae*, *Roseburia hominis*, and *Enterocloster bolteae* were enriched in the LNM group (Table 2). To identify genera and species with greater biological relevance, we also applied linear discriminant analysis (LDA) using the LEfSe tool. The results identified *Ruminococcus*, *Blautia*, and *Mediterraneibacter*, along with their species *Ruminococcus gnavus* and *Blautia wexlerae*, as biomarkers for the NLNM group. In contrast, *Hallella*, *Megasphaera*, and *Veillonella atypica* were identified as biomarkers for the LNM group (Figures 3A,B). These findings suggest that significant alterations occur in gut microbiome composition during the progression of pancreatic cancer, particularly involving *Blautia wexlerae* and *Ruminococcus gnavus*.

**Table 2:** Top 30 differentially abundant microbial species between the LNM and NLNM groups.

Taxon	Mean (LNM_F)	Mean (NLNM_F)	Log2 FC	*P*-value	Adjusted *P*-value*
*Klebsiella pneumoniae*	8914284.891	480327.9811	4.2140	1.552E-06	4.073E-05
*Phocaeicola plebeius*	92148.349	1326702.017	−3.8477	2.090E-08	1.351E-06
*Roseburia hominis*	627579.6661	93804.96373	2.7421	2.079E-06	5.279E-05
*Bifidobacterium pseudocatenulatum*	38368.93839	578329.7989	−3.9139	9.005E-08	4.211E-06
*Enterocloster bolteae*	543127.9671	33047.92909	4.0387	1.133E-12	4.474E-10
*Klebsiella variicola*	498930.622	30300.86765	4.0414	6.800E-07	2.083E-05
*Blautia wexlerae*	56430.15989	376463.1345	−2.7380	7.172E-08	3.590E-06
*Klebsiella quasipneumoniae*	286276.8458	22945.0548	3.6412	3.437E-06	8.089E-05
*Veillonella parvula*	235871.4854	42904.45509	2.4588	7.901E-04	7.805E-03
*Roseburia intestinalis*	405817.2722	123229.2501	1.2778	6.678E-03	4.215E-02
*Ruminococcus gnavus*	863800.9903	250969.1377	−1.7973	1.778E-04	2.290E-03
*Blautia obeum*	50139.72749	116066.7401	−1.2109	9.744E-04	9.199E-03
*Sutterella wadsworthensis*	131134.8817	23651.38358	2.4711	2.694E-03	2.082E-02
*Anaerostipes hadrus*	24101.04084	110990.2619	−2.2033	1.391E-05	2.731E-04
*Bilophila wadsworthia*	85337.2128	67396.65028	1.4981	7.663E-03	4.640E-02
*Klebsiella aerogenes*	105757.2719	67950.97278	3.8514	1.749E-07	7.062E-06
*Bacteroides salyersiae*	78488.40539	29169.4709	1.4280	8.309E-03	4.960E-02
*Coprococcus comes*	30334.10463	60761.0379	−1.0022	2.099E-03	1.671E-02
*Enterocloster aldenensis*	78621.44051	23045.111	3.0138	4.633E-08	2.473E-06
*Bacteroides eggerthii*	62453.9122	21619.18879	1.5305	2.086E-03	1.666E-02
*Dialister invisus*	185678.6867	262020.2333	2.3055	4.921E-04	5.449E-03
*Klebsiella oxytoca*	155539.9762	19670.31585	3.3833	1.570E-06	4.073E-05
*Dialister hominis*	73059.8813	1033.013116	6.1441	1.063E-11	2.099E-09
*Enterocloster clostridioformis*	469318.7399	41121.72916	1.8138	2.454E-05	4.495E-04
*Klebsiella michiganensis*	359665.114	12095.94194	1.9542	7.416E-03	4.539E-02
*Dialister massiliensis*	59657.32939	904.520752	7.8963	5.413E-22	1.924E-18
*Coprobacter fastidiosus*	12654.13813	41201.29105	−1.7031	7.418E-03	4.539E-02
*Megamonas funiformis*	21992.10237	43754.40738	−3.1353	7.047E-07	2.141E-05
*Veillonellaceae bacterium*	52290.91808	1007.064194	5.6984	3.656E-12	9.995E-10
*Klebsiella quasivariicola*	46231.10198	1842.213927	4.6494	1.135E-07	4.920E-06

*P-values were adjusted for multiple testing using Benjamini and Hochberg’s FDR. An adjusted *p*-value of < 0.05 was considered statistically significant.

### Differences in fecal metabolites between the LNM and NLNM groups

Given the complex interactions between the gut microbiota and host–microbe co-metabolism, we conducted non-targeted metabolomics analysis of the fecal samples to assess changes in fecal metabolites during lymph node metastasis in pancreatic cancer. Non-targeted metabolome sequencing identified 4,967 features in negative ion mode (NIM) and 3,544 features in positive ion mode (PIM), with 729 and 587 metabolites annotated, respectively.

**Figure 4:**
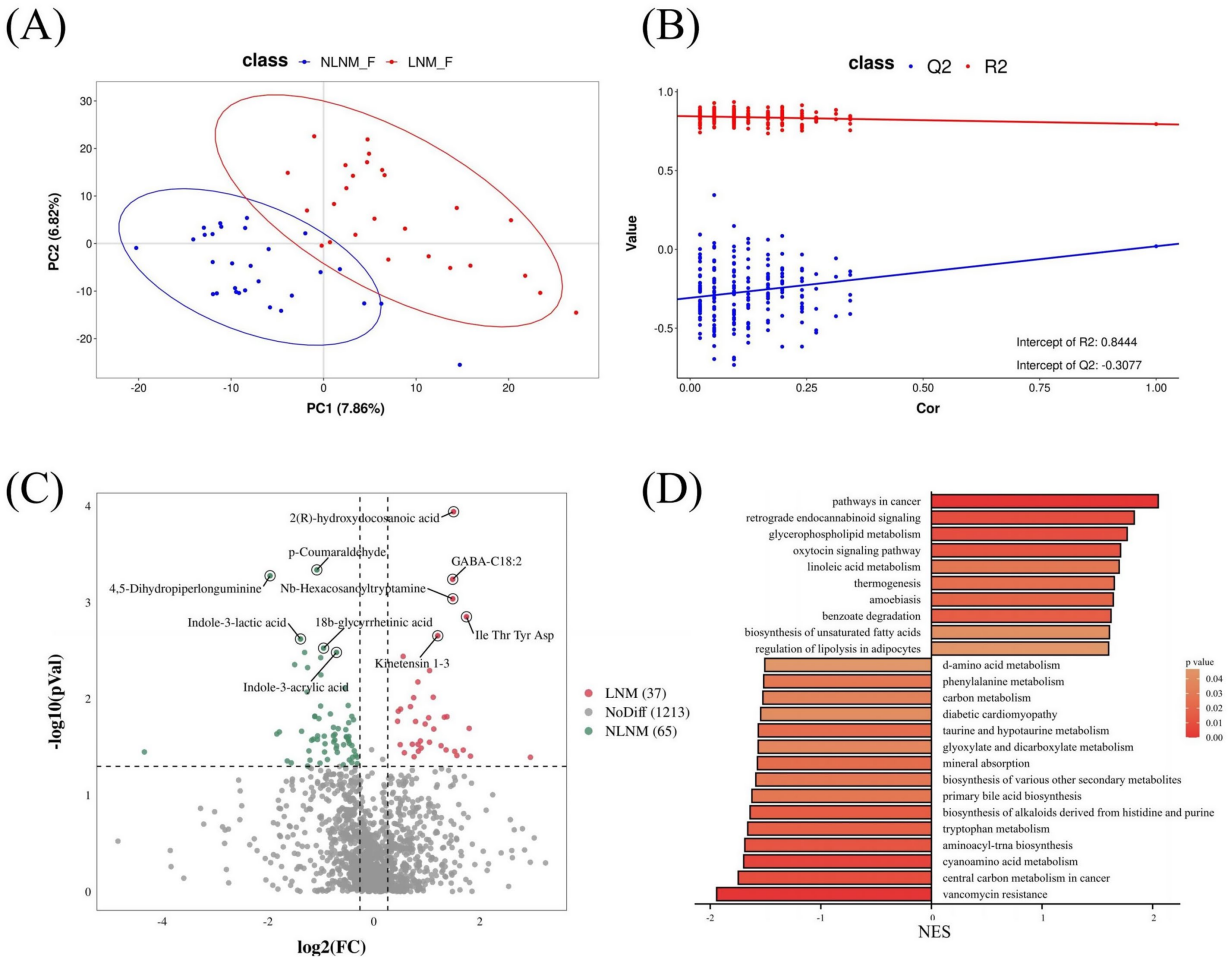
Comparative metabolomics analysis revealed alterations in gut metabolites between the LNM and NLNM groups. Partial least squares–discriminant analysis (PLS-DA) of gut metabolites from both groups **(A)**. PLS-DA permutation test demonstrating model robustness (*R*^2^ = 0.8444, *Q*^2^ = −0.3077) **(B)**. Volcano plot demonstrating changes in metabolites between the LNM and NLNM groups. The *x*-axis indicates log2-transformed fold changes of gut metabolite abundances, and the *y*-axis denotes log10-transformed *p*-values **(C)**. Gene set enrichment analysis (GSEA) reveals differential metabolic pathways between the two groups **(D)**.

The PLS-DA model demonstrated a clear separation in metabolomic composition between the LNM and NLNM groups (Figures 4A,B). Further analysis identified 92 differential metabolites. Of these, 37 metabolites were significantly increased in the LNM group, while 65 metabolites were significantly decreased. The top three significantly increased metabolites in the LNM group were 2(R)-hydroxydocosanoic acid, GABA-C18:2, and Nb-Hexacosanoyltryptamine, whereas p-Coumaraldehyde, 4,5-Dihydropiperlonguminine, indole-3-lactic acid (3-ILA), 18β-glycyrrhetinic acid, and indole-3-acrylic acid (3-IA) were significantly decreased in the LNM group (Figure 4C). Notably, among the significantly reduced metabolites, 3-ILA and 3-IA are both gut microbiota-derived tryptophan metabolites, which have recently been reported to possess tumor-suppressive properties (Zhang et al., 2023). Their marked depletion in the LNM group suggests a potential role in pancreatic cancer progression and lymph node metastasis.

GSEA revealed distinct enrichment patterns of gut microbiota-derived metabolites between the two groups. The NLNM group exhibited enrichment in pathways related to aminoacyl-tRNA biosynthesis, cyanoamino acid metabolism, tryptophan metabolism, primary bile acid biosynthesis, and carbon metabolism. In contrast, the LNM group showed predominant enrichment in pathways related to cancer, glycerophospholipid metabolism, and retrograde endocannabinoid signaling (Figure 4D).

### Correlation analysis of key bacterial species, metabolites, and peripheral blood immune cells

**Figure 5:**
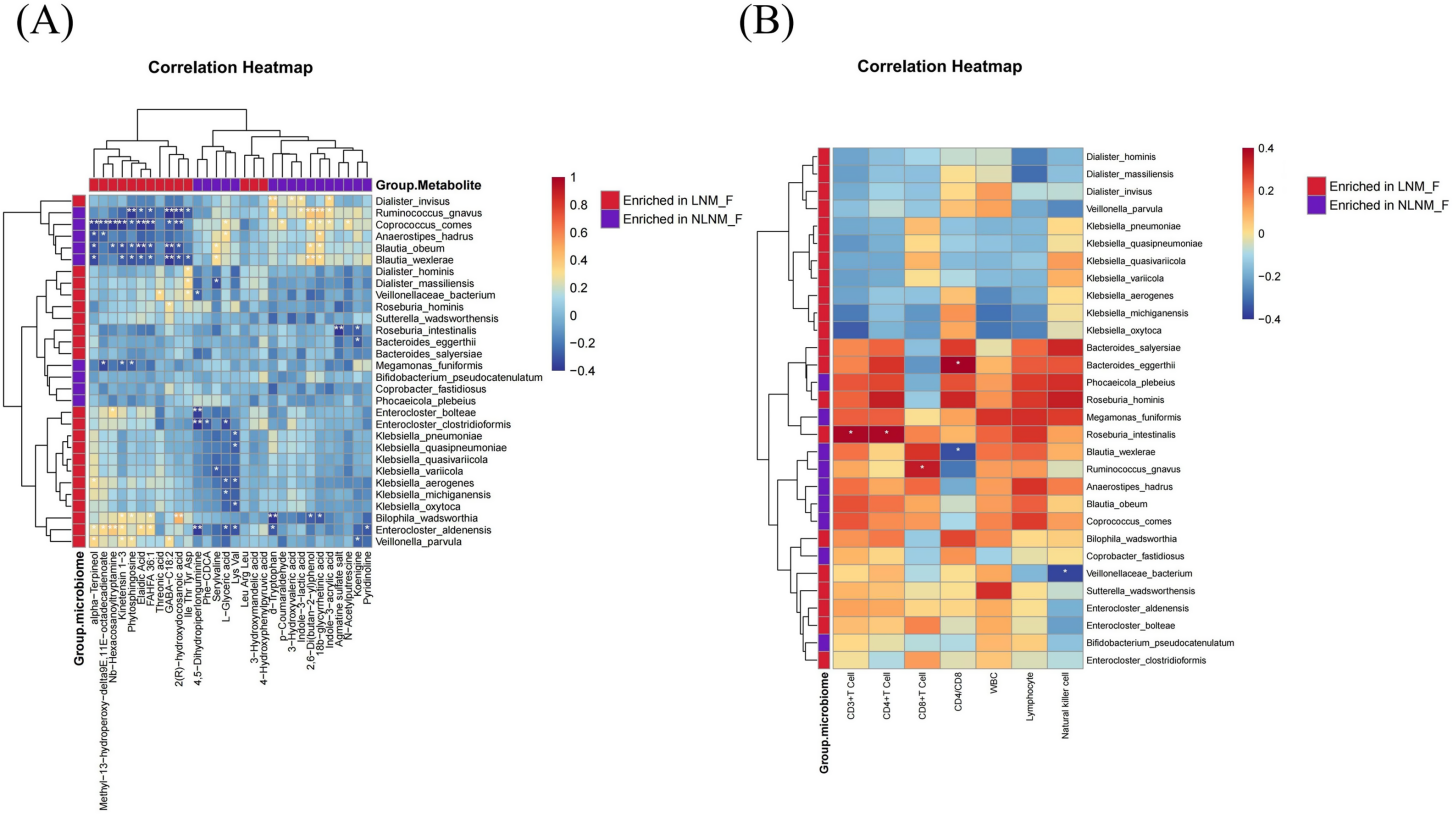
Correlation analysis of the gut microbiota, gut metabolites, and peripheral blood immune cells. Heatmap showing the correlations between the top 30 differential bacterial species and the top 30 differential metabolites **(A)**. Heatmap showing the correlations between the top 30 differential bacterial species and peripheral blood immune cells **(B)**. The red color indicates species enriched in the LNM group, and the purple color indicates species enriched in the NLNM group. Statistical significance was indicated as follows: *p < 0.05, **p < 0.01, and ***p < 0.001.

We performed a correlation analysis of the top 30 differential bacterial species and the top 30 differential metabolites, and the results revealed that the differentially enriched bacterial species in the LNM and NLNM groups were positively correlated with the respective enriched metabolites in each group. In contrast, the differentially enriched bacterial species in the LNM and NLNM groups showed negative correlations with metabolites that were more enriched in the opposite group (Figure 5A). Moreover, we found that the abundance of *Ruminococcus gnavus* was positively correlated with the levels of 3-ILA and 3-IA. We hypothesized that *Ruminococcus gnavus* may influence the expression of key rate-limiting enzymes involved in tryptophan metabolism, particularly those producing indole-3-acrylic acid, or enhance the activity of microbial communities that produce indole-related metabolites, thereby affecting the abundance of these metabolites.

Previous studies have shown that the gut microbiota can activate the systemic immune system and is closely linked to the body’s immune response (Yu et al., 2022; Spencer et al., 2019). Therefore, we further analyzed the relationship between peripheral blood immune cell levels and the gut microbiota. Peripheral blood immune cell counts were measured before surgery in 34 patients, with 13 patients in the LNM group and 21 in the NLNM group. The exploratory correlation network analysis revealed that *Ruminococcus gnavus* was positively correlated with peripheral CD8^+^ T cells. In addition, *Blautia wexlerae* showed a negative correlation with the CD4/CD8 ratio. Conversely, among the species enriched in the LNM group, *Veillonellaceae bacterium* exhibited a negative correlation with NK cells. Furthermore, *Bacteroides eggerthii* was positively correlated with the CD4/CD8 ratio, while *Roseburia intestinalis* was positively associated with CD4^+^ T cells (Figure 5B). These exploratory findings suggest a potential link between the gut microbiota, systemic immune status, and lymph node metastasis, indicating that gut microbes may influence lymphatic dissemination through the modulation of systemic immune responses.

### Construction of a preoperative lymph node metastasis prediction model based on the gut microbiota and metabolites

**Figure 6:**
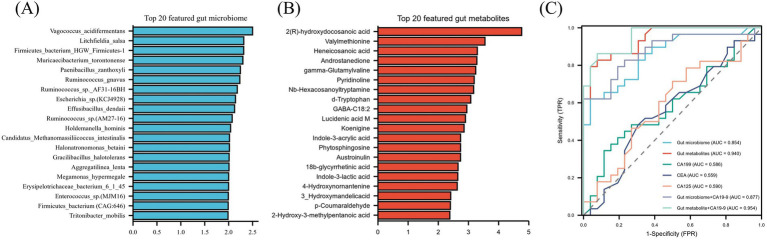
Predictive biomarkers and performance evaluation of classifiers for lymph node metastasis prediction. Featured gut microbiome **(A)** and gut metabolites **(B)** identified for predicting lymph node metastasis using the random forest model, ranked by mean decrease in accuracy. ROC curves evaluating the performance of classifiers in predicting lymph node metastasis, including the gut microbiome, gut metabolites, CA19-9, and CA125 **(C)**.

A random forest algorithm was employed to construct predictive classifiers for lymph node metastasis. To identify the most informative features, microbial species and metabolites were ranked according to their mean decrease in accuracy, reflecting their relative importance in the model. The top 20 microbial features and the top 20 metabolite features with the highest mean decrease in accuracy were selected as biomarkers for model construction (Figures 6A,B). The ROC curves revealed that the gut microbiome classifier had an AUC of 0.854 (95% CI: 0.755–0.953), while the gut metabolite classifier achieved an AUC of 0.940 (95% CI: 0.884–0.996). As CA19-9 and CA125 have been previously reported to be associated with pancreatic cancer progression (Liu et al., 2016; Raza et al., 2024; Liu et al., 2015), their prediction performances were also analyzed, yielding AUC values of 0.586 (95% CI: 0.432–0.739) and 0.590 (95% CI: 0.435–0.745), respectively (Figure 6C), both significantly lower than those of the gut microbiome and gut metabolite classifiers. When the gut microbiome and metabolite classifiers were combined with CA19-9, their prediction performance improved markedly. The gut microbiome + CA19-9 model achieved an AUC of 0.887 (95% CI: 0.784–0.969), while the gut metabolite + CA19-9 model reached an AUC of 0.954 (95% CI: 0.908–1.000) (Figure 6C).

### No significant differences in oral microbiome composition between the LNM and NLNM groups in pancreatic cancer

We also investigated differences in oral microbiome composition between the LNM and NLNM groups. Due to the exclusion of three oral samples that failed quality control, a total of 25 samples from the LNM group and 27 samples from the non-LNM group were included in the analysis. Rarefaction curves based on the Shannon index reached a plateau in both groups, indicating sufficient sequencing depth (Supplementary Figure S1B). *α*-diversity (Simpson index, *p* = 0.99) and *β*-diversity analysis (Bray–Curtis PCoA*, p* = 0.787) revealed no significant differences between the two groups (Supplementary Figures S3A,B). At the genus level, both groups were dominated by *Streptococcus*, *Neisseria*, *Veillonella*, *Haemophilus*, and *Leptotrichia* (Supplementary Figure S3C). LEfSe analysis did not identify any significantly different bacterial taxa between the groups. In summary, our data suggest that the oral microbiome remains largely unchanged during the process of lymph node metastasis in pancreatic cancer.

## Discussion

In this study, we primarily characterized the gut microbiome and its metabolites in patients with lymph node metastasis using metagenomic sequencing and metabolomics analysis. Our analysis revealed, for the first time, the relationship between changes in the gut microbiome and metabolites and LNM in pancreatic cancer. During the process of lymph node metastasis in pancreatic cancer, significant alterations in both the gut microbiome and metabolites were observed. However, no significant differences were detected in the oral microbiome. In terms of differential gut microbiota, metabolites, and metabolic pathways, genera such as *Ruminococcus*, *Blautia*, and *Mediterraneibacter*, along with their species such as *Blautia wexlera* and *Ruminococcus gnavus*, as well as tryptophan metabolites 3-ILA and 3-IA, were significantly reduced in the LNM group, and the activity of the tryptophan metabolic pathway was also markedly decreased in this group. In contrast, *Hallella*, *Megasphaera*, and *Veillonella atypica* were significantly upregulated. Furthermore, based on fecal microbiome and metabolite features, we preliminarily constructed a predictive model for lymph node metastasis in pancreatic cancer, which demonstrated high accuracy, with AUC values reaching 0.854 and 0.940, respectively. Our study provides valuable insights into alterations in the gut microbiome and metabolites during pancreatic cancer progression and highlights potential avenues for microbiome- or metabolite-based diagnostic and therapeutic strategies.

*Ruminococcus gnavus* is a Gram-positive, obligate anaerobic bacterium that has recently been reclassified under the genus *Mediterraneibacter* (Togo et al., 2018). In comparisons between patients with pancreatic cancer and healthy individuals, species with similar functional and metabolic characteristics to *Ruminococcus gnavus*, such as *Ruminococcus bromii* and *Ruminococcus bicirculans*, were reported to be enriched in the feces of healthy individuals (Nagata et al., 2022). Therefore, we speculate that *Ruminococcus* sp. may exert a protective effect against the development and progression of pancreatic cancer. In our study, *Ruminococcus gnavus* was positively correlated with peripheral blood CD8^+^ T cells. Previous studies have reported that *Ruminococcus gnavus* residing in colorectal cancer tissues degrades lyso-glycerophospholipids, a lipid that inhibits CD8 + T cell activity, thereby maintaining the immune surveillance function of CD8 + T cells and inhibiting tumor growth in colorectal cancer (Zhang et al., 2023). Furthermore, oral administration of this *Ruminococcus* sp. to hamsters provided complete protection against SARS-CoV-2 infection through the activation of CD8 + T cell-mediated immunity (Wang et al., 2024). In addition, *Blautia wexlerae*, another species identified as significantly associated with the NLNM group by both DESeq2 and LEfSe analyses, was negatively correlated with the peripheral blood CD4/CD8 T cell ratio. Recent studies have demonstrated a strong association between the gut microbiota and host immune responses, suggesting that gut microbes can influence disease progression and therapeutic outcomes by modulating immune activity (Routy et al., 2018; Gopalakrishnan et al., 2018). Therefore, it is plausible that the gut microbiota may inhibit lymph node metastasis in pancreatic cancer by activating the host immune system, particularly through the enhancement of CD8^+^ T cell activity. However, due to the limited sample size, the analysis of the association between the gut microbiota and immune phenotypes should be considered exploratory. Future *in vivo* experiments are needed for further validation.

3-IA and 3-ILA are primarily derived from tryptophan in the colon, where they are metabolized by the symbiotic gut microbiota through degradation and fermentation into indole metabolites. *In vitro* and *in vivo* studies have shown that 3-ILA significantly inhibits the proliferation of colon cancer cells and reduces tumor volume (Sugimura et al., 2021). Further research by Han et al. demonstrated that 3-ILA exerts its anti-tumor effects by targeting the nuclear receptor RAR-related orphan receptor γt (RORγt) to inhibit T helper 17 cell differentiation, thereby downregulating the IL-17 signaling pathway (Han et al., 2023). In addition, recent studies have suggested that 3-ILA enhances the enrichment of H3K27ac around the IL12a enhancer region and promotes the production of IL12a by dendritic cells (DCs), which, in turn, activates the anti-tumor activity of CD8^+^ T cells (Zhang et al., 2023). In pancreatic cancer, the indole analog of 3-ILA and 3-IA, indole-3-acetic acid (3-IAA), can be oxidized by myeloperoxidase to generate reactive oxygen species (ROS). 3-IAA, in combination with chemotherapy drugs, downregulates ROS-degrading enzymes, leading to the accumulation of ROS and reduced autophagy in cancer cells, thereby improving the efficacy of chemotherapy (Tintelnot et al., 2023). Based on these findings, we hypothesize that the depletion of these tryptophan-derived metabolites could potentially contribute to a tumor-promoting microenvironment. Future studies should investigate whether supplementation with 3-IA or 3-ILA can mitigate metastatic progression and evaluate their potential clinical applications, such as serving as adjuvant therapeutic agents.

Predicting lymph node metastasis in pancreatic cancer has long been a challenging task, with current methods primarily relying on conventional imaging techniques. Our study found significant changes in the gut microbiome and metabolites during lymph node metastasis in pancreatic cancer. Based on these gut characteristics, we built a non-invasive prediction model for lymph node metastasis in pancreatic cancer using machine learning algorithms, achieving diagnostic performance comparable to that of radiomics-based approaches. After integrating CA19-9, the predictive performance of both the gut microbiome and gut metabolite classifiers was further improved, with AUC values reaching 0.877 and 0.954, respectively. In recent years, the advancement of radiomics has also created new opportunities for predicting lymph node metastasis. Tang et al. successfully constructed a lymph node metastasis prediction model using machine learning algorithms combined with 37 ultrasonographic radiomics features, achieving an AUC of 0.85 (Tang et al., 2024). In addition, Fu et al. developed a modified multiview-guided two-stream convolutional network model to predict preoperative lymph node status, incorporating CT radiomics features, CA125, age, and radiologists’ assessments, with an AUC of 0.815 (Fu et al., 2023). Looking ahead, combining the visual clarity of imaging techniques with gut microbiome and metabolite features could further enhance the accuracy of lymph node metastasis prediction, thereby optimizing diagnostic and treatment strategies for pancreatic cancer.

This study has several limitations. First, while our analysis revealed significant changes in the gut microbiome structure and metabolite profiles during the progression of lymph node metastasis in pancreatic cancer, the causal relationship between microbial and metabolic disturbances, immune cell alterations, and pancreatic cancer progression remains unclear. Future research should involve *in vivo* experiments, such as fecal microbiota transplantation or metabolite-based interventions. Second, this study was conducted at a single center with a relatively small sample size, which may restrict the external validity of our findings. Factors such as regional differences, dietary habits, and sequencing platforms could potentially influence gut microbiome and metabolite profiles. Therefore, further validation in large-scale, multi-center studies is essential to confirm the robustness and generalizability of the proposed models. Finally, long-term follow-up studies will be valuable to determine whether the identified microbial and metabolic markers can serve as reliable predictors of lymph node metastasis and even patient prognosis in clinical practice.

## Conclusion

This study revealed distinct gut microbial and metabolite signatures associated with lymph node metastasis in pancreatic cancer. Key microbial species, such as *Ruminococcus gnavus* and *Blautia wexlerae*, were found to be depleted in the LNM group, whereas *Veillonella atypica* was enriched. Notably, the relative abundance of *Ruminococcus gnavus* was positively correlated with peripheral CD8^+^ T cells, suggesting that gut microbiota dysbiosis may contribute to an immunosuppressive microenvironment. Furthermore, the downregulation of tryptophan-derived metabolites (3-IA and 3-ILA) and related metabolic pathways indicates potential microbial and metabolic dysregulation linked to metastatic progression. The predictive models constructed from gut microbiota and metabolite profiles achieved strong discriminatory performance in identifying LNM status. Collectively, these findings provide associative evidence linking gut microbial and metabolic alterations to pancreatic cancer metastasis and highlight their potential as non-invasive biomarkers for clinical prediction. Further mechanistic and functional studies are warranted to determine whether these gut components play a causal role in mediating lymph node metastasis and their interactions with the host immune system.

## Data Availability

The raw sequence data reported in this paper have been deposited in the Genome Sequence Archive (Genomics, Proteomics & Bioinformatics 2021) in National Genomics Data Center (Nucleic Acids Res 2022), China National Center for Bioinformation / Beijing Institute of Genomics, Chinese Academy of Sciences (GSA-Human: HRA010831) that are publicly accessible at https://ngdc.cncb.ac.cn/gsa-human/. Analysis scripts generated in this study are available upon reasonable request.

## Footnotes

1
https://hmdb.ca

2
https://www.genome.jp/kegg
