# Supplementary material for: Integrative metagenomic and metabolomic profiling identifies gut microbial and metabolite signatures associated with lymph node metastasis in pancreatic cancer
Source: Front Microbiol. 2025 Dec 12;16:1706084. doi: 10.3389/fmicb.2025.1706084 (PMC12742467; doi:10.3389/fmicb.2025.1706084)
Supplement: Supplementary file 1 [file Data_Sheet_1.docx]

**Figure S1**


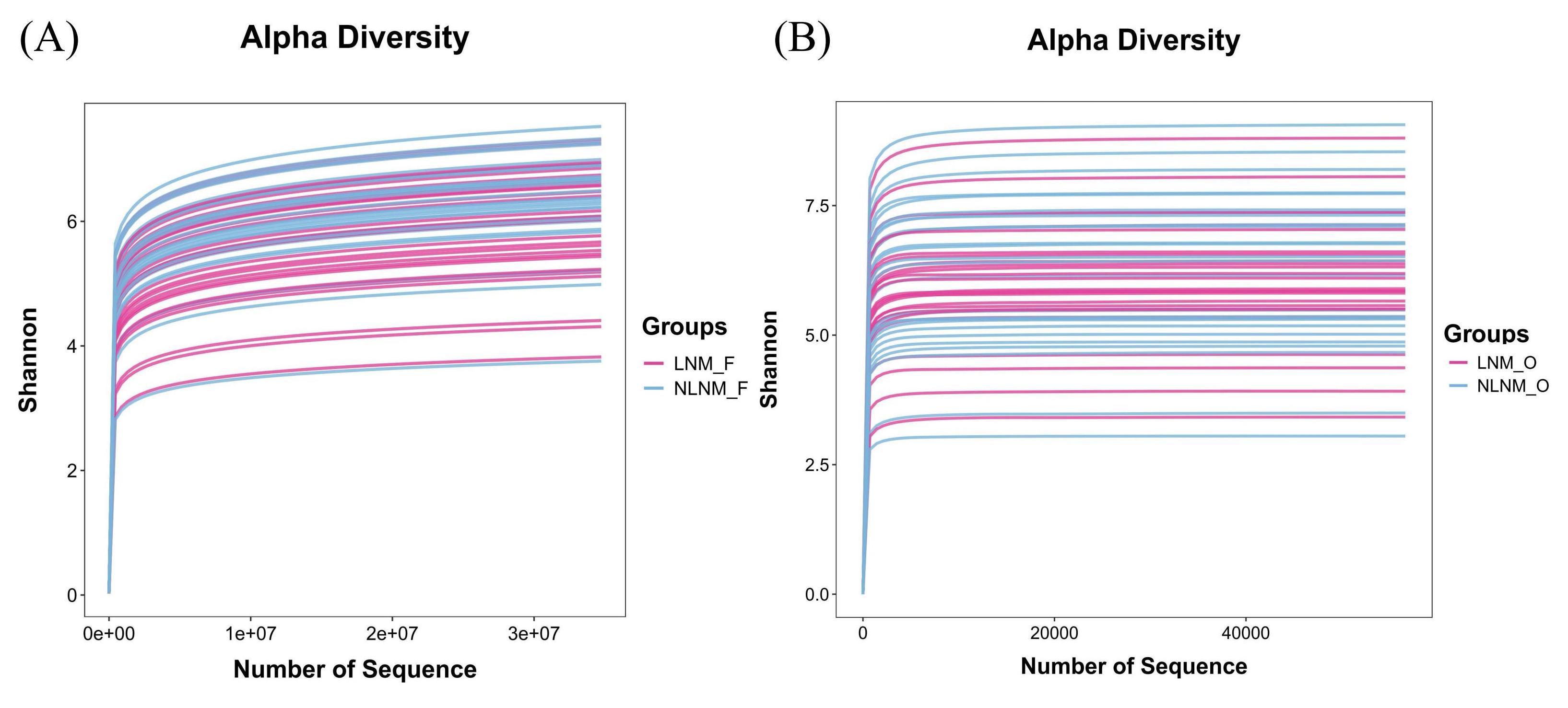


**Figure S1. Rarefaction curves of fecal and oral microbiomes in LNM and NLNM.** The rarefaction curves of fecal (A) and oral (B) samples from LNM and NLNM groups based on the Shannon index were analyzed to demonstrate the stability of the sequencing data.

**Figure S2**


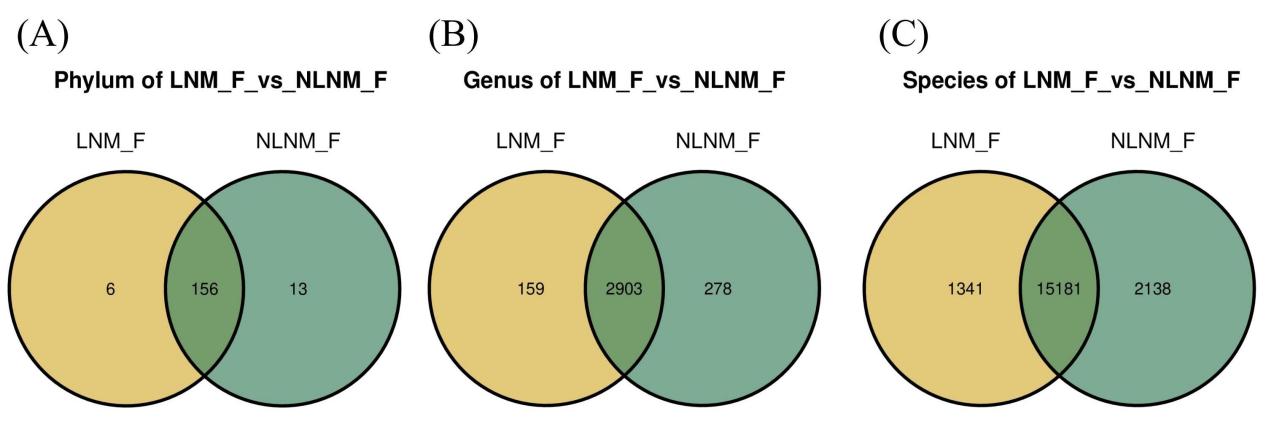


**Figure S2. Venn diagrams of fecal microbiomes in LNM and NLNM**. Venn diagrams showed that LNM and NLNM groups shared 156 phyla (A), 2903 genera (B), and 15181 species (C).

**Figure S3**


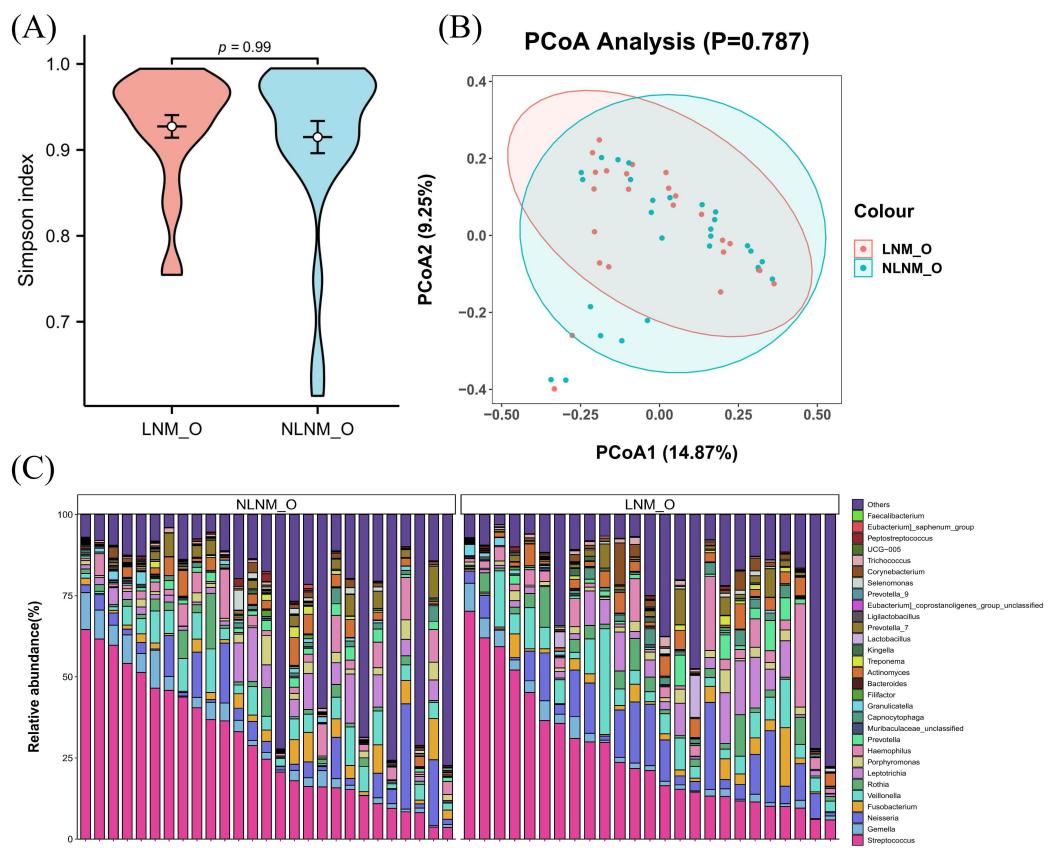


**Figure S3.** **Diversity and Taxonomic Composition Analysis of Oral Microbiome Between LNM and NLNM Groups**. (A) The Simpson index and (B) Bray-Curtis PCoA analysis showed no significant differences between the LNM and NLNM groups. (C) A stacked bar plot displaying the top 30 genera in each sample.
